# *Epimedium brevicornu* Maxim. extract activates natural killer cells against hepatocellular carcinoma via the cGAS-STING pathway

**DOI:** 10.3389/fphar.2025.1681650

**Published:** 2025-11-21

**Authors:** Lu Liu, Xiaoyan Zhan, Xianling Wang, Jincai Wen, Caiping He, Xiaoyan Chen, Yuanyuan Guo, Xueting Wang, Liu Li, Haibo Cheng, Zhaofang Bai, Xiaohe Xiao

**Affiliations:** 1 School of Pharmacy, Chengdu University of Traditional Chinese Medicine Chengdu, Chengdu, China; 2 Senior Department of Hepatology, Chinese PLA General Hospital, Beijing, China; 3 Military Institute of Chinese Materia, Chinese PLA General Hospital, Beijing, China; 4 National Key Laboratory of Kidney Diseases, Beijing, China; 5 School of Medicine, Nankai University, Tianjin, China; 6 The First Clinical Medical College of Nanjing University of Chinese Medicine, Nanjing, China; 7 Jiangsu Collaborative Innovation Center of Traditional Chinese Medicine in Prevention and Treatment of Tumor, Nanjing, China; 8 State Key Laboratory for Quality Ensurance and Sustainable Use of Dao-di Herbs, Beijing, China

**Keywords:** NK cell, *Epimedium brevicornu* Maxim. extract (EPE), hepatocellular carcinoma, immunotherapy, STING

## Abstract

**Background:**

*Epimedium brevicornu*: Maxim., a traditional Chinese botanical drug, has shown significant therapeutic effects against hepatocellular carcinoma (HCC), with its flavonoid compounds exhibiting anti-HCC activity through various mechanisms. However, it remains unclear whether the anti-HCC effects of *Epimedium* are mediated through the activation of natural killer (NK) cells.

**Methods:**

The impact of EPE on NK cell activity was assessed via Enzyme-Linked Immunosorbent Assay (ELISA) and flow cytometry. A co-culture model of NK-92 cells with K562 target cells was constructed to evaluate EPE-enhanced NK cytotoxicity using Calcein-AM release assay. A murine HCC subcutaneous xenograft model was employed to demonstrate EPE-targeted NK cell activation against HCC *in vivo*. NK1.1 cell depletion experiment was conducted to further confirm the NK cell-dependent anti-tumor mechanism. Finally, Western blotting, flow cytometry, molecular docking, Cellular thermal shift assay (CETSA) and Drug affinity responsive target stability assay (DARTS) were used to elucidate the underlying molecular mechanisms of EPE in activating NK cell.

**Results:**

EPE significantly promoted the synthesis and release of IFN-γ, Granzyme B, and Perforin in NK-92 cells and increased the expression of the activating receptor NKG2D on the NK-92 cell and human primary NK cells surface. Treatment of human primary NK cells with EPE increased the proportion of the CD56^bright^CD16^dim^ and CD56^dim^CD16^bright^ NK cell subsets. Calcein release assays demonstrated that EPE enhanced the cytotoxic activity of NK cells against K562 cells. Studies utilizing a murine model of subcutaneous HCC xenografts confirmed that EPE effectively inhibited tumor growth, promoted tumor cell death, and significantly elevated the levels of the NK cell activation markers IFN-γ and CD107a in the spleen and tumor tissues of HCC-bearing mice. NK1.1 cell depletion experiment proved that depletion of NK1.1 cells significantly attenuated the anti-HCC effect of EPE, further demonstrating that the EPE exerts its anti-tumor action by specifically targeting and activating NK cells *in vivo*. Mechanistic studies revealed that EPE promoted IFN-β release in NK cells, significantly increased the phosphorylation levels of STING and IRF3. Concurrently, immunofluorescence results indicated that EPE significantly upregulated the expression level of p-STING protein in tumor-infiltrating NK cells within the tumor tissues of HCC mice. High Performance Liquid Chromatograph (HPLC) revealed that 4H-1-Benzopyran-4-one, Epimedin A1, Epimedin A, Epimedin B, Epimedin C, Icariin, Baohuoside Ⅰwere compounds with higher content in EPE, and there is a strong binding ability between Epimedin C and STING. Moreover, Epimedin C decrease the thermostability of STING but increase the resistance to protein-digesting enzymes.

**Conclusion:**

EPE exerts anti- HCC effects by activating NK cells through the activation of the cGAS-STING signaling pathway. This suggests that EPE may serve as a potential immunotherapeutic agent, offering novel therapeutic perspectives for the treatment of HCC.

## Graphical Abstract

**Figure:**
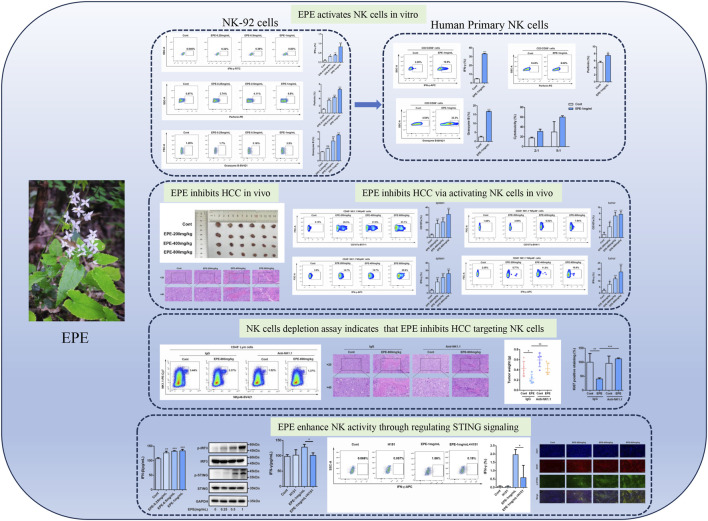

## Highlights

- EPE promotes the activation of NK cells *in vitro*.
- EPE inhibits HCC progress through enhancing NK cells activity *in vivo*.
- NK depletion weakens the role of EPE in suppressing HCC *in vivo*.
- EPE inhibits HCC progress through enhancing NK cells by regulating cGAS-STING pathway.

## 1 Background

Natural Killer (NK) cells, as central effector cells of the innate immune system, have emerged as a cutting-edge focus in tumor immunotherapy in recent years, owing to their unique “pre-sensitization-independent” killing mechanism and role in tumor immunosurveillance (Laskowski et al., 2022). Unlike traditional tumor immunotherapies, such as T-cell therapies, which rely on the recognition of specific antigens, the cytotoxic mechanism of NK cells is independent of tumor antigen presentation (Myers and Miller, 2021). Instead, NK cells dynamically recognize and eliminate tumor cells exhibiting downregulated MHC-I molecules or abnormal stress signals through a balance between surface activating and inhibitory receptors (Parham and Moffett, 2013). Consequently, NK cells possess the distinct advantage of rapid tumor cell recognition and killing, making them particularly suitable for targeting tumor types characterized by significant immune tolerance and evasion. With advancements in gene-editing technologies, chimeric antigen receptor natural killer (CAR-NK) cell therapy has evolved into a groundbreaking emerging therapeutic strategy. Genetic engineering techniques are used to modify NK cells to express chimeric antigen receptors (CARs) targeting tumor-specific antigens. Combining this with pre-activation using cytokines such as IL-15 or IL-21 not only enhances their persistence and proliferative capacity but also improves their ability to infiltrate tumors (Luo et al., 2025; Maalej et al., 2023). Simultaneously, the immunosuppressive nature of the tumor microenvironment (TME) often suppresses NK cell function (Llovet et al., 2024; Yang et al., 2023). Therefore, reversing the immunosuppressive state of the TME has become a major research priority. Enhancing NK cell function within the TME can significantly amplify their anti-tumor effects. These technological advances open new avenues for treating solid tumors, particularly in malignancies like hepatocellular carcinoma (HCC) with prominent immunosuppressive microenvironments, offering promising therapeutic strategies and approaches.

HCC represents the third leading cause of cancer-related mortality worldwide, with treatment outcomes remaining particularly limited in advanced stages (Huang et al., 2023; Llovet et al., 2024). Current systemic therapies, such as sorafenib and PD-1/PD-L1 inhibitors, although widely used clinically, demonstrate objective response rates below 20% and are frequently associated with the development of resistance (Llovet et al., 2024). The therapeutic challenge in HCC stems from its complex TME, characterized by pronounced immunosuppressive features. These include the infiltration of regulatory T cells (Tregs) and myeloid-derived suppressor cells (MDSCs), alongside the secretion of immunosuppressive factors such as TGF-β and IL-10. These immunosuppressive mechanisms severely compromise the efficacy of immunotherapies by dampening the host immune system’s anti-tumor response (Cai et al., 2024; Liu et al., 2023). Consequently, overcoming the immunosuppressive barriers within the TME of HCC and activating/amplifying anti-tumor immunity constitute major research priorities. Studies indicate that activated NK cells can not only effectively penetrate the fibrous capsule of HCC and remodel the TME but also enhance immune responses through the secretion of cytokines like Interferon-γ (IFN-γ) (Xiao et al., 2024). Nevertheless, NK cells within the TME often exhibit functional impairments, manifesting as exhaustion, senescence, and suppression, which significantly limit their therapeutic efficacy against HCC (Zhang et al., 2020). Prolonged exposure to tumor antigens and immunosuppressive factors particularly leads to a progressive decline in NK cell function and a consequent attenuation of their anti-tumor activity (Li Y et al., 2024). Therefore, developing strategies to enhance the function and tumoricidal capacity of NK cells specifically within the HCC context has emerged as a critical research direction in contemporary immunotherapy.

*Epimedium brevicornu* Maxim., a traditional Chinese botanical drug used for “tonifying kidney yang and strengthening tendons/bones,” contains bioactive compounds icariin and icaritin, which have demonstrated multi-target antitumor effects (Chen et al., 2023; Haoyue et al., 2025; Mou et al., 2024; Yu et al., 2020). In HCC models, *E. brevicornu* Maxim. extract (EPE) can activate ERK/ULK1/NCOA4-mediated iron autophagy, thereby enhancing the survival conditions of HCC-afflicted rats and mitigating liver damage and carcinogenesis (Han et al., 2025). Notably, studies indicate that icariin promotes dendritic cell maturation and CD8^+^ T cell activation, thereby enhancing the efficacy of anti-PD-1 therapy (Haoyue et al., 2025). However, systematic studies are lacking on whether EPE directly enhances NK cell cytotoxicity or reverses HCC microenvironment-induced NK cell dysfunction. Elucidating the molecular mechanisms by which EPE extract regulates NK cell activity could provide an experimental foundation for developing Traditional Chinese Medicine (TCM) approaches to activate immune cells, potentially offering a strategy to overcome the current response bottlenecks in HCC immunotherapy.

This study represents the first systematic investigation into the efficacy of EPE in inhibiting HCC through activating NK cell. We established activation assessment systems using both the NK-92 cell line and human primary NK cell *in vitro*. The impact of EPE on NK cell activity was systematically evaluated by determining key activation markers via ELISA and flow cytometry. Furthermore, the specific molecular mechanisms underlying EPE-induced NK cell activation were investigated using Western blotting and Elisa. The findings from this research are expected to elucidate the underlying mechanisms by which EPE targets and activates NK cells, offering novel therapeutic strategies for combination therapies against HCC.

## 2 Methods and materials

### 2.1 Mice

Male C57BL/6J (18–22 g, 6–8 weeks old) were purchased from Sibeifu Beijing Biotechnology Co. Ltd. (SPF Biotechnology, Beijing, China). The mice were acclimatized in a controlled environment with a temperature range of 22 °C–24 °C, humidity levels maintained at 50%–60%, and a 12-h light/dark cycle. They had unrestricted access to food and water throughout the acclimatization period. All animal procedures were conducted in strict adherence to the ethical guidelines and welfare regulations approved by the Ethics Committee of The Fifth Medical Center of the PLA General Hospital (approval number: IACUC-2024-0004).

### 2.2 Antibodies and reagents

*Epimedium* was purchased from Chunfeng Pharmaceutical (Beijing, China). Human Fc Receptor Blocking Solution (422301, Biolegend, US), Purified anti-mouse CD16/CD32 (FMU16/32-02-100, 4A Biotech, China), FITC anti-human IFN-γ Antibody (506504, Biolegend, US),APC anti-human IFN-γ Antibody (502512, Biolegend, US),PE anti-human Perforin Antibody (308106, Biolegend, US), Brilliant Violet 421™ anti-human/mouse Granzyme B Recombinant Antibody (396414, Biolegend, US), FITC anti-human CD3 (981002, Biolegend, US), PE/Cyanine7 anti-human CD56 (985912, Biolegend, US), FITC anti-human CD16 (980112, Biolegend, US), PE anti-human CD314 (NKG2D) Antibody (320806, Biolegend, US), Brilliant Violet 711™ anti-human CD107a (LAMP-1) Antibody (328640, Biolegend, US), APC anti-human CD107a (LAMP-1) Antibody (328620, Biolegend, US), PE anti-human TNF-α (986802, Biolegend, US), FITC anti-mouse CD45 Antibody (157214, Biolegend, US), PE/Cyanine7 anti-mouse NK-1.1 Antibody (156514, Biolegend, US), Brilliant Violet 421™ anti-mouse CD335 (NKp46) Antibody (137612, Biolegend, US), APC anti-mouse IFN-γ Antibody (505810, Biolegend, US), Brilliant Violet 711™ anti-mouse CD107a (LAMP-1) Antibody (121631, Biolegend, US), TMEM173/STING Polyclonal antibody (19851-1-AP, Proteintech), IRF3 Polyclonal antibody (11312-1-AP, Proteintech), Phospho-TMEM173/STING (Ser366) (AF7416, Affinity), Anti-IRF3 (phospho S386) (ab76493, Abcam), Anti-GAPDH (GB15002-100, Servicebio) and NCR1 (DF7599, affinity) were used.

### 2.3 Cell culture

NK-92 cells were obtained from Pricells (Wuhan, China) and cultured in MEMα medium supplemented with 0.2 mM Inositol, 0.1 mM β-mercaptoethanol, 0.02 mM Folic Acid, 150 U/mL recombinant IL-2, 12.5% human serum (HS), 12.5% fetal bovine serum (FBS), and 1% penicillin/streptomycin (P/S). The cells were maintained under standard conditions, with a gas phase composition of 95% air and 5% CO_2_, at a temperature of 37 °C. K-562 (Pricells, Wuhan, China) were cultured in RPMI 1640 medium (Boster, Wuhan, China) supplemented with 10%FBS and 1% P/S. The cells were maintained in a cell incubator with 5% CO2 and passaged to generations 5-10 for cytotoxicity assays. Hepa1-6 (Pricells, Wuhan, China) were cultured in DMEM medium (Boster, Wuhan, China) supplemented with 10%FBS and 1% P/S. Hepa-1-6 cells were passaged to the 5th-10th generation for subcutaneous tumor implantation.

### 2.4 Human NK cell isolation and cell culture

Blood samples were obtained from healthy donors who had provided written informed consent. The study was approvaled by the Ethics Committee of The Fifth Medical Center of the PLA General Hospital (approval No. KY-2024-7-104-1). Peripheral blood mononuclear cells (PBMCs) were separated using the Lymphoprep™ (Stemcell Technologies). The PBMCs were then cultured at a concentration of 5 × 10^6^ cells per mL in X-VIVO 15 (Lonza) supplemented with 10% heat-inactivated fetal calf serum (FCS, Gibco) and 1% P/S at 37 °C for 18 h. The cells were activated with IL-12 (30 ng/mL, Pricells) and IL-15 (100 ng/mL, Pricells).

### 2.5 High performance liquid chromatograph (HPLC)

EPE was identified by Chengdu Glip Bio-Technology Co., Ltd (Chengdu, China). Briefly, The Waters Arc Premier analysis method for identifying compounds in traditional Chinese medicine (TCM) extracts involves several key steps. Briefly, the EPE is dissolved in an appropriate solvent and pre-processed. Next, the sample is separated using HPLC under optimized conditions. Following separation, mass spectrometry (MS) is employed for online detection and qualitative and quantitative analysis of the compounds. The information of the major compound in EPE is showed in Supplementary Table S1.

### 2.6 Enzyme-linked reaction

NK-92 cells were seeded at a density of 5 × 10^^4^ cells per well in a 96-well plate, with a total volume of 100 μL per well, and incubated overnight in a cell culture incubator. The cells were then treated with different concentrations of EPE (0, 0.25 mg/mL, 0.5 mg/mL, 1 mg/mL) for 24 h. The level of IFN-γ released by the cells was measured using the Human IFN-γ ELISA Kit (Dakewei, Shenzhen, China).

### 2.7 Quantitative real-time PCR

NK-92 cells were seeded at a density of 1 × 10^6^ cells per well in a 12-well plate, with a total volume of 1 mL per well, and incubated overnight in a cell culture incubator. The cells were then treated with different concentrations of EPE (0, 0.25 mg/mL, 0.5 mg/mL, 1 mg/mL) for 18 h. After treatment, the cells were blocked for 6 h, and RNA was extracted using the Trizol method. The RNA was then reverse transcribed into cDNA using a reverse transcription kit (CWBIO, Jiangsu, China). The gene expression was analyzed using the SuperStar Universal SYBR Master Mix (CWBIO, Jiangsu, China) and the Q6 Real-Time PCR System (BioRad). The data were normalized to the actin mRNA levels. The primer sequences are as follows:β-Actin (H), forward: 5′- CAT​GTA​CGT​TGC​TAT​CCA​GGC-3′; reverse: 5′- CTC​CTT​AAT​GTC​ACG​CAC​GAT-3′. IFN-γ (H), forward: 5′- GAG​TGT​GGA​GAC​CAT​CAA​GGA​AG-3′; reverse: 5′- TGC​TTT​GCG​TTG​GAC​ATT​CAA​GTC-3′. Perforin (H), forward: 5′- ACT​CAC​AGG​CAG​CCA​ACT​TTG​C-3′; reverse: 5′- CTC​TTG​AAG​TCA​GGG​TGC​AGC​G-3′. Granzyme B (H), forward: 5′- CGA​CAG​TAC​CAT​TGA​GTT​GTG​CG-3′; reverse: 5′- TTC​GTC​CAT​AGG​AGA​CAA​TGC​CC-3′. NKp46 (H), forward: 5′- CAG​CAA​CTT​GCT​GGA​TCT​GGT​G-3′; reverse: 5′- AGA​CGG​CAG​TAG​AAG​GTC​ACC​T-3′. FasL (H), forward: 5′- GGT​TCT​GGT​TGC​CTT​GGT​AGG​A-3′; reverse: 5′- CTG​TGT​GCA​TCT​GGC​TGG​TAG​A-3′. IFN-γ (M), forward: 5′- CAG​CAA​CAG​CAA​GGC​GAA​AAA​GG-3′; reverse: 5′- TTT​CCG​CTT​CCT​GAG​GCT​GGA​T-3′. Perfroin (M), forward: 5′- ACA​CAG​TAG​AGT​GTC​GCA​TGT​AC-3′; reverse: 5′- GTG​GAG​CTG​TTA​AAG​TTG​CGG​G-3′. Granzyme B (M), forward: 5′- CAG​GAG​AAG​ACC​CAG​CAA​GTC​A-3′; reverse: 5′- CTC​ACA​GCT​CTA​GTC​CTC​TTG​G-3′. NKp46 (M), forward: 5′- TAG​GGC​TCA​CAG​AGG​GAC​ATA​C-3′; reverse: 5′- GTA​GGT​GCA​AGG​CTG​CTG​TTC​T-3′. CD107a (M), forward: 5′- CCA​GGC​TTT​CAA​GGT​GGA​CAG​T-3′; reverse: 5′- GGT​AGG​CAA​TGA​GGA​CGA​TGA​G-3′. Data were presented using the 2^-ΔΔCT method, normalize with the control group.

### 2.8 Flow cytometry

Fluorochrome-conjugated antibodies against human/mouse proteins were obtained from Biolegend. For NK-92 cells and Human Primary NK cell: cells were seeded in 12-well plates at a density of 1 × 10^^6^ cells/mL and cultured overnight. The cells were then treated with different concentrations of EPE for 18 h. After treatment, 1 μg/mL monensin solution (BioLegend, US) was added to each well and incubated for 6 h to block cytokine secretion. The cells were washed 2-3 times with PBS, then incubated at 4 °C with human Fc receptor blocking antibodies for 10 min. Next, the cells were stained at 37 °C for 20 min with the following antibodies: CD3, CD56, CD16, and NKG2D. Following staining, the cells were fixed with fixative (Biolegend, US) at room temperature for 10 min, and then permeabilized with permeabilization solution (Biolegend, US) at room temperature for 15 min. After centrifugation at 2000 rpm for 5 min, intracellular staining was performed for IFN-γ, Perforin, Granzyme B, and TNF-α. Finally, the expression of relevant factors was analyzed using flow cytometry.

For spleen tissue cells: fresh mouse spleens were placed in RPMI-1640 medium containing 2% serum. The tissue was passed through a sterile 40 μm mesh to collect the cell suspension. The cells were centrifuged at 2000 rpm for 5 min, and the supernatant was discarded. The pellet was resuspended in PBS, centrifuged again at 2000 rpm for 5 min, and the supernatant was discarded. The cells were then resuspended in red blood cell lysis buffer (Biolegend, US) and incubated on ice for 5 min. After centrifugation at 2000 rpm for 5 min, the supernatant was discarded, and the cells were resuspended in PBS. The process was repeated once more to wash the cells. The cells were then incubated with CD16/32 (Biolegend, US) antibody at room temperature in the dark for 10 min, followed by centrifugation at 2000 rpm for 5 min and removal of the supernatant. The cells were washed with PBS and resuspended in an antibody mix (CD45-FITC, NK1.1-PE-Cy7, NKp46-BV421) and stained at room temperature in the dark for 20 min. After centrifugation at 2000 rpm for 5 min and removal of the supernatant, the cells were washed once with PBS. The cells were then fixed and permeabilized, followed by intracellular staining with IFN-γ-APC and CD107a-BV711. After centrifugation at 2000 rpm for 5 min, the supernatant was discarded, and the cells were resuspended in 200 μL of PBS. The cell suspension was filtered and analyzed by flow cytometryor tumor tissue cells: fresh mouse tumor tissues were placed in RPMI-1640 medium containing 2% serum. Each sample was digested with 1.5 mL of digestion solution (0.2 mg/mL DnaseⅠ(Solarbio, Beijing, China), 1 mg/mL Collagenase IV (Solarbio, Beijing, China), and 0.2 mg/mL Hyaluronidase (Solarbio, Beijing, China) dissolved in 2% FBS RPMI-1640) at 37 °C for 1 h. The cell suspension was then filtered through a sterile 40 μm mesh to collect the cells. The cells were centrifuged at 2000 rpm for 5 min, and the supernatant was discarded. The cell pellet was resuspended in PBS, centrifuged again at 2000 rpm for 5 min, and the supernatant was discarded. Subsequently, CD16/32 antibody was added and incubated at room temperature in the dark for 10 min. After centrifugation at 2000 rpm for 5 min, the supernatant was discarded, and the cells were washed with PBS and resuspended. The subsequent antibody staining steps followed the same protocol as for spleen tissue cells. The results were analyzed using FlowJo V10 software.

### 2.9 CD107a degranulation assay

Briefly, Human Primary NK cells were pretreated with or without EPE for 24 h. Then cells were stimulated with K562 cells at a 1:1 effector-to-target ratio for 4 h in the presence of 1 μg/mL CD107a antibody and 1 μg/mL Monensin. After stimulation, cells were harvested and surface-stained with CD3 and CD56 for 20 min at room temperature in the dark. After staining, the cells were washed and resuspended in PBS for flow cytometry analysis.

### 2.10 Cytotoxicity assay

K562 cells were labeled with 2 μM Calcein-AM (Sigma-Aldrich) for 30 min at 37 °C, protected from light. The labeled target cells were then washed twice with PBS and resuspended in culture medium. Then NK-92 cells, pre-treated or untreated with the EPE, were collected as effector cells and seeded into a 96-well plate at effector-to-target ratios of 2:1 and 5:1, with K562 cells as the target cells for 4 h at 37 °C. After incubation, the supernatants were collected, and fluorescence intensity was measured using a fluorescence microplate reader (excitation: 485 nm, emission: 530 nm). The percentage of target cell lysis was calculated by comparing the fluorescence intensity of the drug-treated NK cell culture wells with that of the untreated NK cell culture wells. Cytotoxicity (%) = 100×(maximum release−spontaneous release)/(experimental fluorescence−spontaneous release).

### 2.11 Hepa1-6 tumor model and drug treatment

The mice were randomly divided into the control group (equal volume of saline solution) and the EPE group (200 mg/kg, 400 mg/kg, 800 mg/kg). The mice are anesthetized with isoflurane and the dorsal flank region is disinfected with 70% ethanol. A27G needle is used to inject 200 μL of the Hepa1-6 cell suspension (approximately 1 × 10^^6^ cells) subcutaneously into the left or right flank of each mouse. Care is taken to avoid injecting into the muscle or causing damage to surrounding tissues. Starting from the third day after tumor implantation, the mice were treated with gavage for 1 month. After 1 month of continuous gavage, the mice were anesthetized with isoflurane, and the spleen and tumors were collected. A portion was fixed with paraformaldehyde for pathological analysis, a portion of fresh tissue was used for flow cytometry analysis, and another portion was used for mRNA detection.

### 2.12 NK1.1 cell depletion assay

For NK1.1 cell depletion, starting from day 3 post-subcutaneous tumor cell implantation, 250 µg of Asialo GM1 Polyclonal Antibody, Functional Grade eBioscience™ (16-6507-39, Thermo Fisher) was administered intraperitoneally every 3 days for a total of three injections.

### 2.13 Hematoxylin and eosin staining

Tumor tissue fixated using paraformaldehyde was dehydrated with alcohol. The tissue is then cleared in xylene, embedded in paraffin, and sectioned into thin slices. After rehydration, the sections are stained with hematoxylin, which stains cell nuclei blue, and eosin, which stains cytoplasm and extracellular matrix pink. The sections are then dehydrated, cleared again, and mounted with a cover slip. Finally, observe the tumor pathology under a microscope.

### 2.14 Immunohistochemistry

Tumor tissue was excised and embedded in paraffin, then sectioned and deparaffinized, followed by hydration. Next, the sections are treated with an antigen retrieval solution (such as citrate buffer) to unmask the antigens. The Ki67-specific primary antibody (1:100) is incubated with the sections, typically overnight at 4 °C. Subsequently, a secondary antibody conjugated with HRP is applied, and the DAB chromogenic reaction is performed, resulting in brown precipitates. Finally, hematoxylin counterstaining is carried out, and the Ki67-positive cell percentage is observed under a microscope to assess tumor proliferation.

### 2.15 Immunofluorescence

Tumor tissue sections were excised and subjected to deparaffinization and hydration. Next, antigen retrieval is performed using an antigen retrieval solution to unmask the antigens. The sections are then incubated with p-STING (1:100) and NCR1 (1:100) primary antibodies, typically overnight at 4 °C. Following this, secondary antibodies conjugated with fluorescent dyes corresponding to the primary antibodies are applied, using different wavelengths of fluorescence for labeling. Finally, the sections are observed under a fluorescence microscope to assess the co-localization of the two markers and analyze the expression of STING and NCR1.

### 2.16 Western blotting analysis

The protein samples were separated by SDS-PAGE, transferred onto a PVDF membrane, and probed with primary antibodies targeting the protein of interest. Antibodies used were as follows: TMEM173/STING Polyclonal antibody, IRF3 Polyclonal antibody, Phospho-TMEM173/STING (Ser366), Anti-IRF3 (phospho S386), Anti-GAPDH. Following incubation with appropriate secondary antibodies conjugated to HRP, protein bands were visualized chemiluminescent detection. The signal intensity of the bands was quantified using ImageJ software.

### 2.17 Molecular docking

The design of small molecules and the STING protein was carried out using ChemBio3D Ultra 14.0 and Pymol2.3.0, respectively. Protein binding sites were predicted using POCASA 1.1, and docking was performed with AutoDock Vina 1.1.2. For STING, the following parameters were set: center x = 113.0, center y = 130.5, center z = 126.3, with a search space of size_x = 60, size_y = 60, and size_z = 60 (cell spacing set to 0.375 Å). All other parameters were kept at their default settings. To analyze the interaction patterns of the docking results, PyMOL 2.3.0 and Ligplot V2.2.8 were used.

### 2.18 Cellular thermal shift assay (CETSA)

The NK-92 cells were collected, following freezed and thawed with liquid nitrogen 2-3 times, then centrifuged at 1,2000 rpm to collect the supernatant. Next split into two equal portions. One portion was incubated with compound (100 μM), while the other was treated with DMSO at 37 °C for 30 min. Both groups were then further divided into 7 equal samples and heated for 3 min at temperatures of 45 °C, 48 °C, 51 °C, 54 °C, 57 °C, 60 °C, and 63 °C. After heating, cell supernatants were collected by centrifugation at 12,000 rpm and 4 °C for 10 min. The samples were then analyzed using the immunoblotting method.

### 2.19 Drug affinity responsive target stability assay (DARTS)

The cell supernatant was obtained by lysing the cells on ice using M-PER buffer (Thermo Fisher Scientific, USA), which was then diluted with 10 × TNC buffer (50 mM Tris–HCl, pH = 8.0, 50 mM NaCl, 10 mM CaCl_2_). The supernatant was divided into two portions: one portion received compound (100 μM) and the other was treated with DMSO. Both samples were incubated for 1 h at room temperature. After incubation, Pronase (1:2000, Roche, Switzerland) was added and the mixture was incubated for another 30 min at 37 °C. The protein samples were analyzed using the immunoblotting method.

### 2.20 Statistical analysis

Statistical analyses were conducted using GraphPad Prism 6.02. For differential analysis, one-way ANOVA and t-tests were applied. All data were obtained from at least three independent biological replicates. Statistical significance was considered when P < 0.05, with ^*^P < 0.05, ^**^P < 0.01, and ^***^P < 0.001 indicating different levels of significance.

## 3 Results

### 3.1 EPE effectively promotes the release of active and toxic factors in NK-92 cells

**FIGURE 1:**
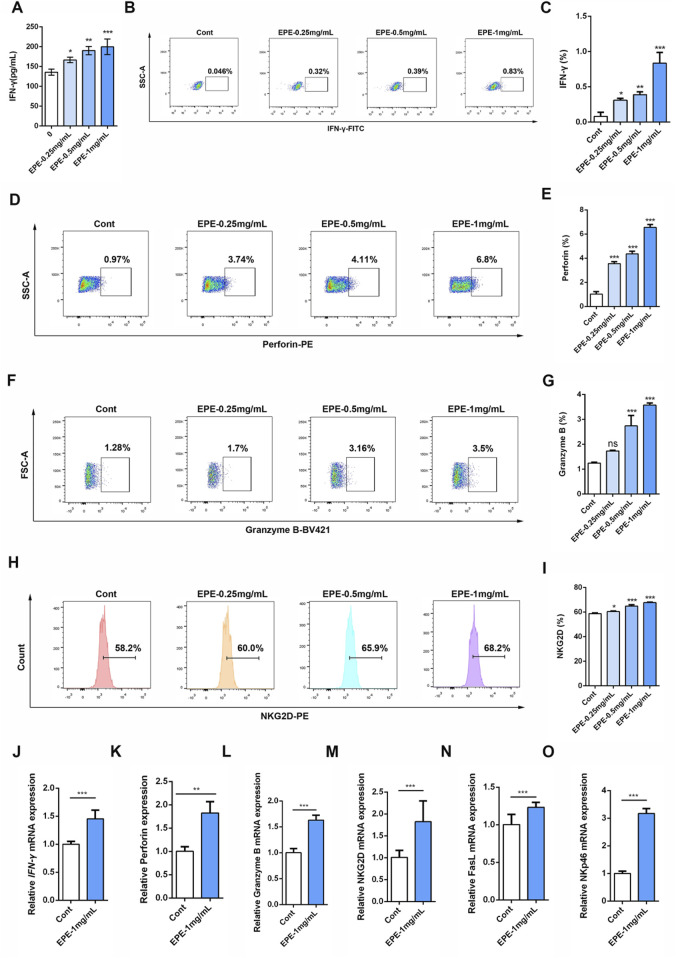
EPE promotes the release of active factors in the NK-92 cell. **(A)** The NK-92 cells were seeded in a 96-well plate, and then intervened with different concentrations of EPE (0, 0.25 0.5, 1 mg/mL) for 24 h. ELISA kit was used measure the levels of IFN-γ. **(B–I)** Representative flow plots of IFN-γ **(B)**, perfroin **(D)**, Granzyme B **(F)** and NKG2D **(H)** and quantification of IFN-γ **(C)**, Perforin **(E)**, Granzyme B **(G)** and NKG2D **(I)** in NK-92 cells treated with varying concentrations of EPE (0, 0.25, 0.5, and 1 mg/mL) for 18 h followed by an additional 6 h treatment with monensin solution. Numbers adjacent to outlined areas indicate percent the mean fluorescence intensity of IFN-γ, Granzyme B, Perforin and NKG2D. **(J–O)** The mRNA levels of IFN-γ **(J)**, Perforin **(K)**, Granzyme B **(L)**, NKG2D **(M)**, FasL **(N)** and NKp46 **(O)** in NK-92 cells treated with varying concentrations of EPE (0, 0.25, 0.5, and 1 mg/mL) for 18 h followed by an additional 6 h treatment with 1 μg/mL monensin solution was detected using qRT-PCR method. Data are represented as mean ± SEM. n = 3. ^∗^ *p* < 0.05, ^∗∗^ *p* < 0.01, ^∗∗∗^ *p* < 0.001.

IFN-γ is a potent multifunctional cytokine which is secreted primarily by activated NK cells (Huot et al., 2023). To explore the effect of EPE on NK cell activity, this study systematically evaluated its impact using ELISA and flow cytometry. The results demonstrated that, compared to the control group, EPE significantly promoted the secretion of IFN-γ in NK cell at concentrations of 0.25 mg/mL, 0.5 mg/mL, and 1 mg/mL (Figures 1A–C). Previous studies have shown that activation of NK cell increase the synthesis and release of Perforin and Granzyme B, thereby enhancing their ability to kill tumor cells (Prager et al., 2019; Rubin et al., 2017). Based on these findings, this study further investigated the effects of EPE on the synthesis and release of Perforin and Granzyme B in NK cell. Flow cytometry results indicated that the expression levels of Perforin and Granzyme B in NK cell from the treatment groups were significantly higher than those in the untreated group (Figures 1D–G). Additionally, NKG2D, an important activating receptor on the surface of NK cell, is responsible for recognizing stress molecules on the surface of tumor and infected cells (Peng et al., 2022). The study further examined the effect of EPE on NKG2D expression in NK cell using flow cytometry, and the results showed that EPE significantly increased the expression level of the NKG2D receptor on the surface of NK cell (Figures 1H,I). Furthermore, qRT-PCR results further confirmed that EPE induced an increase in the gene transcription levels of NK cell activity-related factors, including IFN-γ, Granzyme B, Perforin, NKG2D, FasL, and NKp46 (Figures 1J–O). In conclusion, this study provides preliminary evidence that EPE can effectively enhance NK cell activity, offering experimental support for its potential application as an immunomodulatory agent.

### 3.2 EPE promotes the activity of human primary NK cell *in vitro*

**FIGURE 2:**
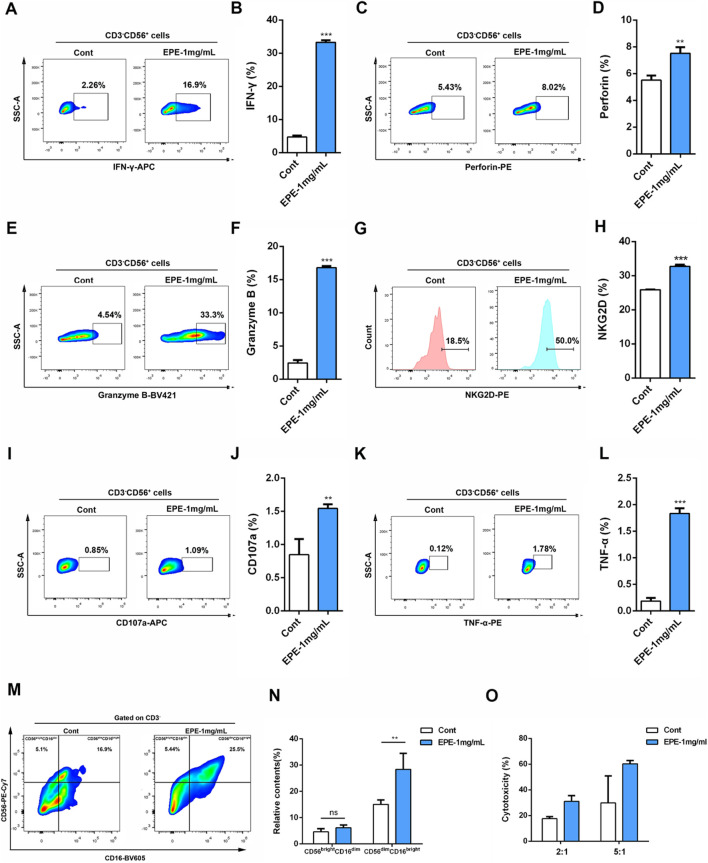
EPE activates human primary NK cell. **(A–H)** Flow-cytometry analysis of the production of IFN-γ **(A)**, Perforin **(C)**, Granzyme B **(D)** and NKG2D **(G)** and the quantification of IFN-γ **(B)**, Perforin**(D)**, Granzyme B **(F)** and NKG2D **(H)** in human primary NK cell un-treated or treated with EPE (1 mg/mL) for 18 h followed by an additional 6 h treatment with 1 μg/mL monensin solution. **(I)** Flow-cytometry analysis of the production of CD107a in human primary NK cell untreated or treated with EPE (1 mg/mL) for 24 h, following co-cultured with K562 cells for 4 h. **(J)** The quantification of CD107a. **(K)** Flow-cytometry analysis of the production of TNF-α in human primary NK cell. **(L)** The quantification of TNF-α. **(M,N)** Flow-cytometry analysis of the production of CD56^bright^CD16^dim^ and CD56^dim^CD16^bright^ cells and the quantification of CD56^bright^CD16^dim^ and CD56^dim^CD16^bright^ cells in human primary NK cell un-treated or treated with EPE (1 mg/mL) for 18 h followed by an additional 6 h treatment with 1 μg/mL monensin solution. **(O)** Cytotoxicity of NK cell untreated or treated for 24 h with (1 mg/mL) and incubated for 4 h with K562 cells (effector: target = 2:1 or 5:1) was measured by Calcein-AM assay. Cytotoxicity (%) = 100×(maximum release−spontaneous release)/(experimental fluorescence−spontaneous release). Data are represented as mean ± SEM. n = 3. ^∗^ *p* < 0.05, ^∗∗^ *p* < 0.01, ^∗∗∗^ *p* < 0.001.

Based on the activation effect of EPE on the NK-92 cell line, this study further investigated the influence of EPE on human primary NK cell activity. We analyzed the impact of EPE on the secretion of IFN-γ, Granzyme B, and Perforin by human primary NK cell using flow cytometry. The results showed that, compared to the control group, EPE-treated human primary NK cell exhibited a significantly enhanced ability to produce IFN-γ, Granzyme B, and Perforin (Figures 2A–F). Moreover, the expression level of NKG2D was also significantly higher in EPE-treated NK cell compared to untreated cells (Figures 2G,H). Lysosomal-associated membrane protein 1 (CD107a) is transported to the cell surface during the cytotoxic process of NK cell (Rubin et al., 2017). Based on the ability of EPE to increase the release of Granzyme B and Perforin in NK cell, we co-cultured NK cell with K562 liver cancer cells and assessed the expression of CD107a on the NK cell surface to evaluate NK cell toxicity and activity. The results showed that, compared to the control group, the expression of CD107a degranulation was significantly increased in EPE-treated NK cell after co-culture with K562 cells (Figures 2I,J). Meanwhile, it also enhances the ability of NK cell to release tumor necrosis factor-α (TNF-α), a cytokine used to assess NK cell activity (Mujal et al., 2025) (Figures 2K,L). Interestingly, EPE also promoted an increase in the proportion of CD56^bright^ CD16^dim^ NK cell and CD56^dim^ CD16^bright^ NK cell in human primary NK cell (Figures 2M,N). These two subsets of cells are involved in cytokine secretion and cytotoxic killing, respectively (Cooper et al., 2001). Furthermore, we established a co-culture model of NK-92 cells and K562 cells, and applied the calcein-AM release assay to examine the impact of EPE on NK cell-mediated tumor cell killing. The study results indicated that, under effector-to-target ratios of 2:1 and 5:1, EPE-treated NK cell significantly enhanced their killing ability against K562 tumor cells (Figure 2O). In conclusion, this study demonstrates that EPE significantly promotes NK cell activity and toxicity, and enhances the cytotoxic capacity of NK cell against tumor cells *in vitro*.

### 3.3 EPE inhibits HCC mice tumor growth through enhancing NK cell activation *in vivo*

**FIGURE 3:**
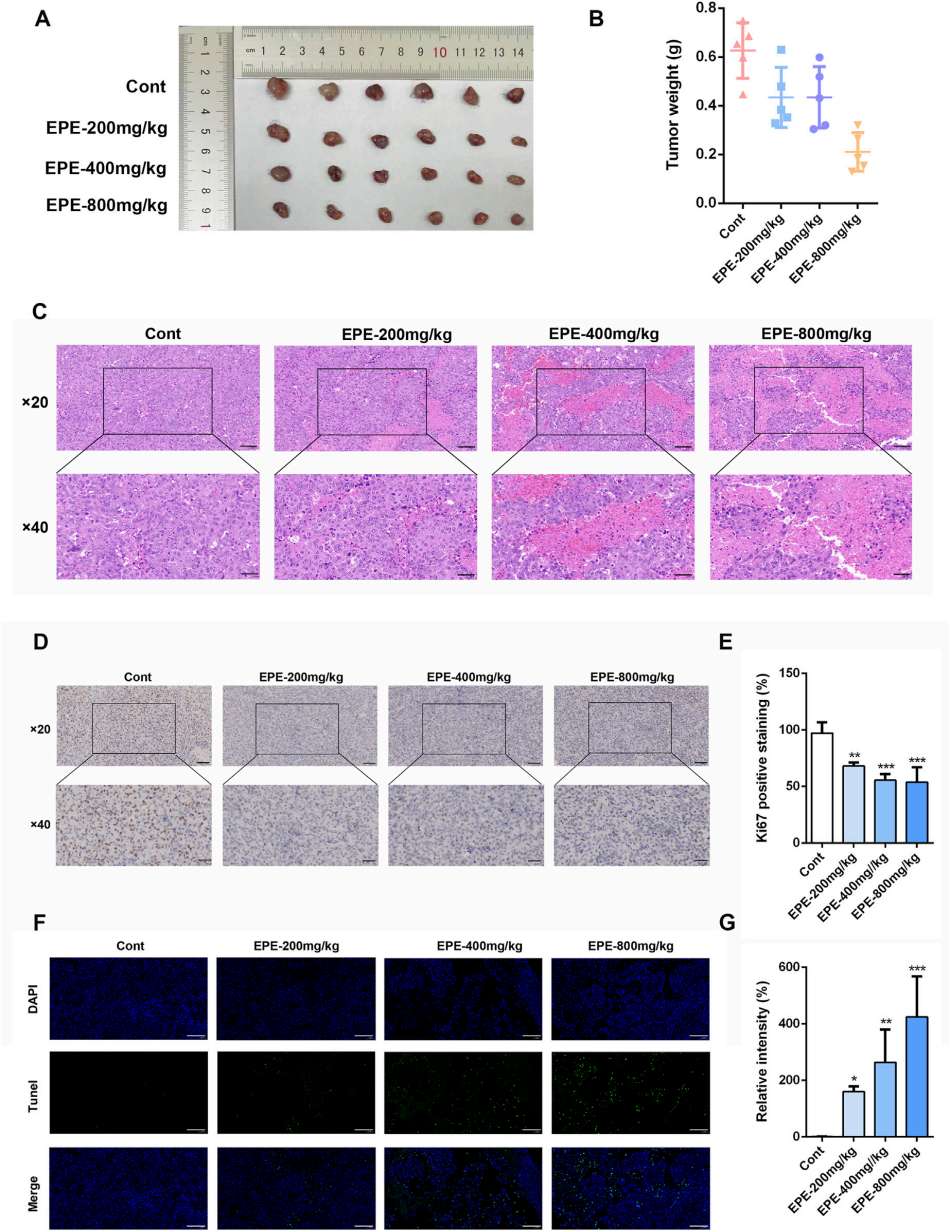
EPE can inhibit the growth of HCC. **(A)** Mice were sacrificed 1 month after subcutaneous inoculation with 2 × 10^^6^ Hepa-1-6 cells. Representative appearance of mouse tumors following intervention with EPE (0, 200, 400, 800 mg/kg). Scale bar = 20 μM or 50 μM. **(B)** Tumor weight of mice in each intervention group. **(C)** Representative pathological images of tumors in mice from each intervention group. **(D)** Representative Ki67 staining images of tumors in mice from the EPE intervention group and the non-intervention group. The darker the brown color, the higher the cell proliferation. Scale bar = 20 μM or 50 μM. **(E)** The quantification of Ki67 expression using ImageJ Pro Plus. **(F)** Representative Tunel staining images of tumors in mice from the EPE intervention group and the non-intervention group. Scale bar = 20 μM. **(G)** The quantification of Tunel using Image J. Data are represented as mean ± SEM. n = 5. ^∗^ *p* < 0.05, ^∗∗^ *p* < 0.01, ^∗∗∗^ *p* < 0.001.

**FIGURE 4:**
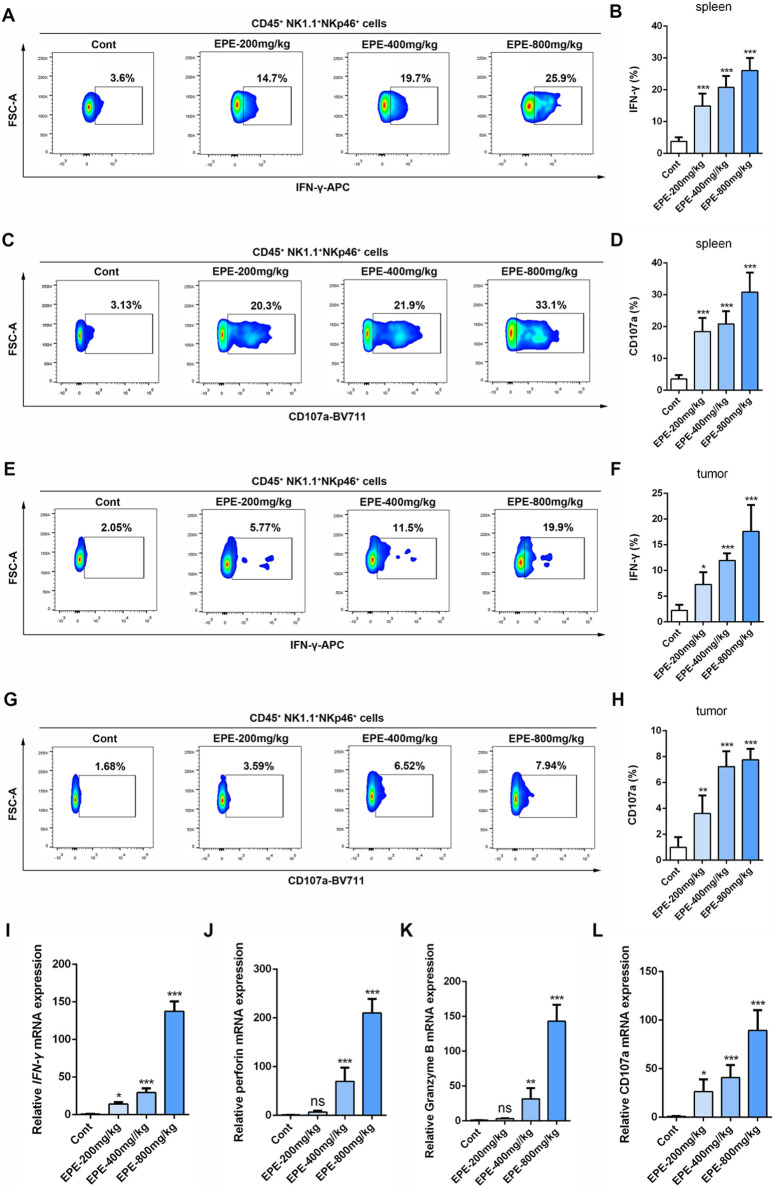
EPE regulates NK cell function to resist HCC. **(A–D)** Flow cytometry analysis of NK cell IFN-γ **(A)** and CD107a **(C)** expression in spleen tissue of tumor-bearing mice. Numbers adjacent to outlined areas indicate percent the mean fluorescence intensity of IFN-γ, CD107a. Quantitative analysis of IFN-γ **(B)** and CD107a **(D)** levels in NK cell. **(E–H)** Flow cytometry analysis of NK cell IFN-γ **(E)** and CD107a **(G)** expression in tumor tissue of tumor-bearing mice. Quantitative analysis of IFN-γ **(F)** and CD107a **(H)** levels in NK cell. **(I–L)** qRT-PCR analysis of IFN-γ, Perforin, Granzyme B and CD107a mRNA levels in tumor tissue were measured. Data are represented as mean ± SEM. n = 5. ^∗^ *p* < 0.05, ^∗∗^ *p* < 0.01, ^∗∗∗^ *p* < 0.001.

This study further investigates whether the anti-HCC effect of EPE is associated with NK cell activation. The results showed that, compared to the control group, EPE at doses of 200 mg/kg, 400 mg/kg, and 800 mg/kg effectively inhibited tumor growth in mice, while also reducing tumor weight (Figures 3A,B). Histopathological analysis revealed a significant increase in tumor necrosis in the EPE-treated group compared to the untreated group (Figure 3C). Ki-67 and Tunel staining results indicated that, compared to the control group, EPE suppressed tumor cell proliferation and simultaneously promoted tumor cell apoptosis (Figures 3D–G). These findings suggest that EPE exhibits anti-HCC effects. NK cell, as one of the main effector cells of the innate immune system, kill tumor cells by releasing cytotoxic molecules (such as CD107a) and secreting cytokines (like IFN-γ). We further assessed the impact of EPE on NK cell activity-related cytokines, IFN-γ and CD107a, in the spleen and tumor tissues of tumor-bearing mice using flow cytometry. The results demonstrated that, following EPE treatment, the levels of IFN-γ and CD107a in both spleen and tumor tissues were significantly elevated compared to the control group (Figures 4A–H). Additionally, qRT-PCR analysis revealed that the mRNA expression levels of IFN-γ, Granzyme B, Perforin, and CD107a were significantly higher in the tumor tissues of EPE-treated mice than in the untreated group (Figures 4I–L). Taken together, these results suggest that EPE exerts its anti-HCC effect by promoting NK cell activity and regulating immune responses.

### 3.4 NK1.1 cell depletion weakens the inhibitory effect of EPE against HCC *in vivo*

**FIGURE 5:**
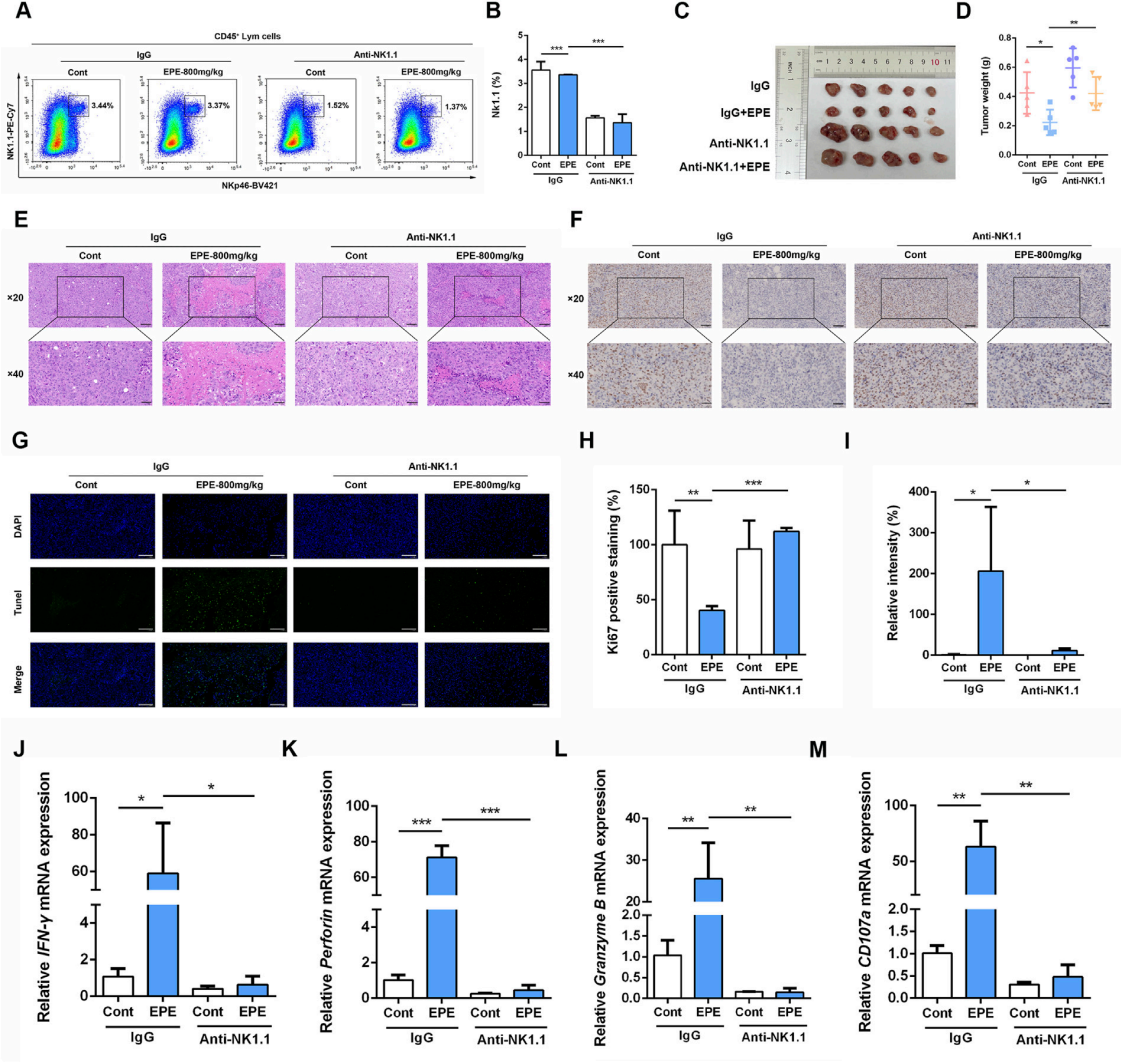
The inhibitory of EPE on HCC is weakened by NK1.1 cell depletion. **(A)** Beginning on day 3 after subcutaneous Hepa-1-6 tumor cell implantation, 250 µg of Asialo GM1 Polyclonal Antibody, Functional Grade eBioscience™ was administered intraperitoneally every 3 days for a total of three injections. After continuous administration for 1 month, the mice were sacrificed. The amount of NK1.1 cells in tumor tissue were measured using flow cytometry. **(B)** The quantification of NK1.1 cells in tumor tissue. n = 3. **(C)** Representative images of tumor in IgG group, IgG plus EPE (800 mg/kg) group, Anti-NK1.1 group and Anti-NK1.1 plus EPE (800 mg/kg) group. **(D)** The analysis of tumor weight is showed. **(E)** Representative images of H&E sections of tumor in each group. Scale bar = 20 μM or 50 μM. **(F)** Representative Ki67 staining images of tumors in each group. Scale bar = 20 μM or 50 μM n = 5. **(G)** Representative Tunel staining images of tumors in each group. Scale bar = 20 μM. **(H)** The quantification of Ki67 positive expression was measured by ImageJ Pro Plus. n = 5. **(I)** The quantification of Tunel positive expression was measured by Image J. n = 5. **(J–M)** qRT-PCR analysis of IFN-γ, Perforin, Granzyme B and CD107a mRNA levels in tumor tissue were measured. n = 3. Data are represented as mean ± SEM. ^∗^ *p* < 0.05, ^∗∗^ *p* < 0.01, ^∗∗∗^ *p* < 0.001.

To further validate the role of NK cell in the anti-HCC effect of EPE, we conducted an NK1.1 cell depletion experiment. Flow cytometry results showed that the number of NK1.1 cell in the tumor tissues of mice in the Asialo GM1 polyclonal antibody intervention group was significantly reduced compared to the untreated group (Figures 5A,B). Confirming the successful establishment of the depletion model. As shown in Figure 4C, after EPE intervention, tumor growth in mice was significantly inhibited. In comparison to the IgG group, the tumor volume in the NK1.1 cell-depleted EPE-treated group was significantly larger (Figure 5C). Additionally, NK1.1 cell depletion weakened the inhibitory effect of EPE on tumor weight in mice (Figure 5D). Histopathological analysis further revealed that NK1.1 cell depletion significantly impaired the tumor cell necrosis induced by EPE (Figure 5E). Ki-67 and Tunel staining results showed that, compared to the EPE-only intervention group, NK1.1 cell depletion markedly reduced the ability of EPE to inhibit tumor cell proliferation and induce tumor cell apoptosis (Figures 5F–I). qRT-PCR analysis confirmed that NK1.1 cell depletion attenuated the EPE-induced elevation of NK activity-related molecule mRNA expression in tumor tissues. The results demonstrated that, compared to the IgG group, EPE significantly increased the mRNA transcription levels of IFN-γ, Granzyme B, Perforin, and CD107a, whereas NK1.1 cell depletion significantly abolished the EPE-induced elevation of these mRNA levels in tumor tissue cells (Figures 5J–M). These findings suggest that the anti-HCC effect of EPE is dependent on NK cell in the tumor tissues of mice.

### 3.5 EPE promotes NK cell activation via regulating the cGAS-STING signaling pathway

**FIGURE 6:**
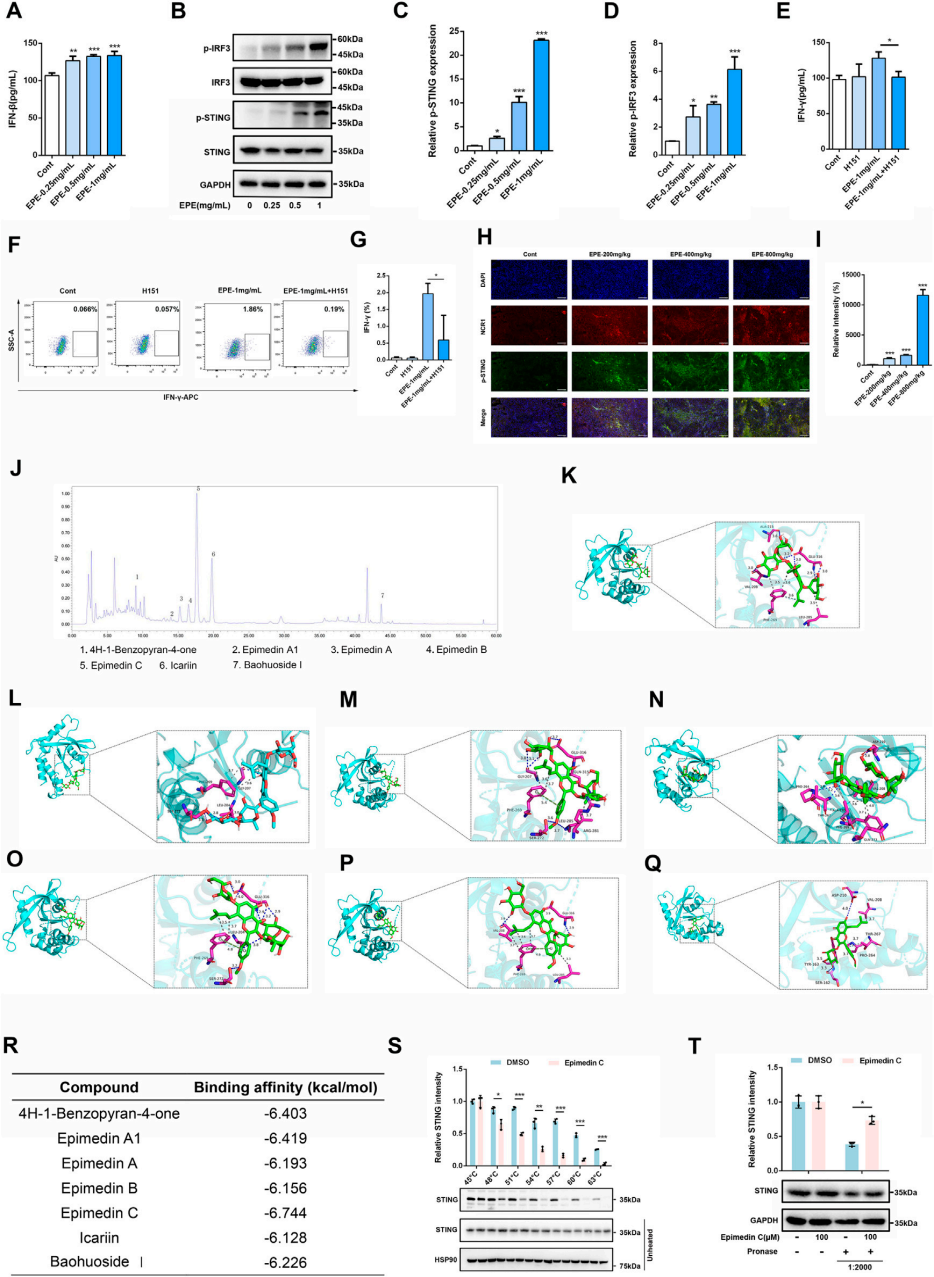
EPE activates NK cell via activating cGAS-STING signaling pathway. **(A)** Elisa analysis of IFN-β levels in NK-92 cells treated with EPE (0, 0.25, 0.5,1 mg/mL) for 24 h. n = 3. **(B)** NK-92 cells were untreated or treated with EPE (0.25, 0.5,1 mg/mL) for 24 h. The expression p-IRF3, IRF3, p-STING, STING was measured using Western blotting. GAPDH as an internal reference. **(C,D)** The quantitative results of the ratio of p-STING and p-IRF3 normalized to their respective total proteins are shown. n = 3. **(E)** Elisa analysis of IFN-γ in NK-92 cells treated with EPE (1 mg/mL), H151 (10 μM) or EPE (1 mg/mL) plus H151 (10 μM) for 24 h. n = 3. **(F)** Flow cytometry analysis of IFN-γ in NK-92 cells treated with EPE (1 mg/mL), H151 (10 μM) or EPE (1 mg/mL) plus H151 (10 μM) for 18 h followed by an additional 6 h treatment with monensin solution. Numbers adjacent to outlined areas indicate percent the mean fluorescence intensity of IFN-γ. **(G)** The quantification of IFN-γ expression in NK-92 cells. n = 3. **(H)** Representative immunofluorescence image of p-STING expression in NK cell of tumor tissues. Blue fluorescence represents the cell nucleu, red fluorescence represents NK cell and green fluorescence represents the p-STING protein **(I)**. The quantification of p-STING in NK cell of tumor tissue using Image J. Data are represented as mean ± SEM. n = 5. **(J)** The compounds of EPE was analyzed by HPLC. **(K–Q)** The binding mode of 4H-1-Benzopyran-4-one, Epimedin A1, Epimedin A, Epimedin B, Epimedin C, Icariin, Baohuoside Ⅰand STING was simulated by molecular docking. **(R)** The binding energy of 4H-1-Benzopyran-4-one, Epimedin A1, Epimedin A, Epimedin B, Epimedin C, Icariin, Baohuoside Ⅰand STING protein was showed in table. **(S)** Epimedin C (100 μM) was incubated with the total protein of NK-92 cells, and then the effect of Epimedin C on the thermal stability of STING was analyzed by CETSA assay. n = 3. **(T)** The Epimedin C (100 μM) was preincubated with total protein of NK-92 cells, DARTS assay was used to measure the effect of Epimedin C on digestive ability of STING resist pronase. n = 3. ^∗^ *p* < 0.05, ^∗∗^ *p* < 0.01, ^∗∗∗^ *p* < 0.001.

Once it has been clarified that EPE can significantly promote the activation phenotype of NK cells, this study need to further investigate the mechanism of EPE in activating NK cell. In the previous experiments, this study found that EPE can promote the release of IFNγ, accordingly, we also conducted ELISA to assess the effect of EPE on the production of Interferon-β (IFN-β) by NK-92 cells. Interestingly, EPE can significantly promote the release of IFNβ, which is an important downstream factor released upon activation of the cGAS-STING signaling pathway. Besides, previous studies have shown that the activation of the intrinsic cGAS-STING signaling pathway could promote NK cell function (Lu et al., 2023). The results showed that EPE treatment significantly increased the production of IFN-β in NK cell (Figure 6A). Subsequently, we used Western blotting to evaluate the impact of EPE on the expression of proteins related to the activation of the cGAS-STING pathway in NK cell. The study revealed that, compared to the control group, EPE treatment significantly increased the phosphorylation levels of Stimulator of Interferon Genes (STING) and Interferon Regulatory Factor 3 (IRF3) in NK cell (Figures 6B–D). To further confirm that EPE modulates NK cell activity via the cGAS-STING signaling pathway, we conducted a reverse pharmacological experiment using the cGAS-STING pathway inhibitor H151. The results showed that the addition of H151 significantly reduced the level of IFN-γ production by NK cell promoted by EPE (Figure 6E). These findings were also validated by flow cytometry (Figures 6F,G). Additionally, we further examined the effect of EPE on the phosphorylation of STING in NK cell within mouse tumor tissues. Immunofluorescence results indicated that, compared to the control group, EPE significantly increased the expression of phosphorylated STING in NK cell within the tumor tissue (Figures 6H,I). These results suggest that EPE activates NK cell through the cGAS-STING signaling pathway both *in vitro* and vivo. After clarifying that EPE may activate NK cell function through the cGAS-STING pathway, it is essential to further identify the effective active compounds and their target specificity for STING activation. Subsequently, an HPLC analysis of EPE was performed (Figure 6J), selecting the seven compounds with the highest relative expression levels for molecular docking with STING (Figures 6K–Q). The results showed that Epimedin C exhibited the strongest binding affinity, with a binding energy of −6.744 kcal/mol. Additionally, Epimedin A1 and 4H-1-Benzopyran-4-one also demonstrated good binding affinity to STING, with binding energies of −6.419 kcal/mol and −6.403 kcal/mol, respectively (Figure 6R). The binding between Epimedin C and STING is primarily mediated by hydrogen bonds and hydrophobic interactions. The amino acids involved in hydrogen bond formation with the small molecules are LEU204, SER272, and GLU316, while PHE269 is involved in hydrophobic interactions. To further validate the targeting of Epimedin C to STING, DARTS and CETSA experiments were performed. As shown in Figures 6S,T, Epimedin C reduced the thermal stability of the STING protein while enhancing its resistance to enzyme digestion, confirming that Epimedin C is the main small molecule compounds in Epimedium that targets and activates STING.

## 4 Discussion

*Epimedium*, a botanical agent extensively employed in TCM, has garnered significant research interest for its anti- HCC properties (Han et al., 2025). Flavonoid compounds within *Epimedium*, particularly icariin, exhibit potent immunomodulatory effects (Haoyue et al., 2025; Zheng et al., 2019). Previous study revealed that icariin possessed synergistic interactions with the PD-1 signaling pathway to enhance anti-tumor efficacy and directly induces apoptosis in HCC cells (Haoyue et al., 2025). Collectively, these findings suggest that EPE may mediate its antitumor activity through modulation of the innate immune system. NK cells, serving as pivotal effector lymphocytes within innate immunity, play an indispensable role in tumor immunosurveillance (Vivier et al., 2024). This function is critically relevant in HCC, a malignancy characterized by a profoundly immunosuppressive TME, where inhibitory signals frequently compromise NK cell functionality (Donne and Lujambio, 2023). Utilizing integrated *in vitro* and *in vivo* experimental approaches, this study systematically elucidates the mechanism by which EPE increases NK cell activity for its effect of anti-HCC through targeted NK cell activation. Crucially, our research demonstrates that EPE significantly potentiates NK cell activity via modulation of the cGAS-STING signaling pathway, underscoring its translational potential as an immunotherapeutic strategy against HCC.

NK cells, as core effector cells of the innate immune system, exert anti-tumor functions through the activation of surface receptors such as NKG2D and the release of cytotoxic molecules, including Granzyme B and Perforin (Christodoulou et al., 2021; Khaleafi et al., 2023). Perforin forms pores on the target cell membrane, allowing Granzyme B to enter the cell and induce apoptosis (Voskoboinik et al., 2015). This study found that EPE significantly increased the expression of NKG2D on the surface of NK-92 cells and human primary NK cells, and promoted the secretion of key effector molecules, such as IFN-γ, Granzyme B, and Perforin, consistent with PCR analysis results. Additionally, the study observed the regulatory effects of EPE on NK cell subsets. In human peripheral blood, NK cells can be divided into several subsets (Hofman et al., 2024). The CD56^^bright^CD16^^dim^ subset accounts for about 10% of peripheral blood NK cells and mainly secretes cytokines, with high IFN-γ expression and strong proliferative ability. The CD56^^dim^CD16^^bright^ subset, which accounts for about 90% of peripheral blood NK cells, has strong cytotoxicity and rich reserves of Granzyme B and Perforin (Freud et al., 2017). The study found that after EPE intervention, the proportions of CD56^^bright^CD16^^dim^ and CD56^^dim^CD16^^bright^ subsets in primary human NK cells were significantly increased, suggesting that EPE has a dynamic regulatory effect on the functional state of NK cells. The dual activation of these two subsets by EPE may constitute a synergistic anti-tumor mechanism, forming a “dual approach” anti-tumor model.

The HCC microenvironment is characterized by a high degree of immune suppression, and the dysfunction of NK cells is a key factor in immune escape (Heinrich et al., 2022; Pan et al., 2023; Xiao et al., 2024). This study used a subcutaneous HCC xenograft mouse model to observe the inhibitory effects of EPE on the tumor phenotype and evaluated its impact on the activity of NK cells in both the tumor and spleen within the animal model. CD107a is a critical marker of NK cell degranulation, directly reflecting NK cell cytotoxic function (Vego et al., 2016). When NK cells recognize and attack target cells, their cytotoxic granules, including Perforin and granzymes, fuse with the cell membrane and release their contents, resulting in the transient exposure of CD107a on the cell surface (Krzewski et al., 2013). Therefore, the expression level of CD107a is an important indicator for assessing NK cell activation and killing capacity. The study found that EPE significantly inhibited tumor growth in mice and notably increased the expression levels of NK cell activation markers, such as IFN-γ and CD107a, in the spleen and tumor tissue of HCC-bearing mice. This suggests that EPE may exert anti-HCC effects by enhancing NK cell activity. Further NK cell exhaustion experiments showed that as NK cell function was depleted, the anti-tumor effects of EPE were significantly diminished, indicating that its anti-tumor effects rely on the activity of NK cells. These findings confirm clinical observations, as the functional impairment of NK cells in the peripheral blood and tumor-infiltrating NK cells in HCC patients is closely associated with poor prognosis. Therefore, EPE may modulate anti-tumor immune responses in the HCC microenvironment by activating NK cell activity.

Subsequently, this study further explored the specific molecular mechanisms by which EPE activates NK cells *in vitro*. First, the secretion of interferon factors was detected using ELISA, and it was found that EPE extract effectively activated the release of IFN-β in a concentration-dependent manner. In recent years, several studies have confirmed that the cGAS-STING pathway has become a hotspot in anti-tumor immunology research (Kwon and Bakhoum, 2020; Li X et al., 2024; Xian et al., 2024). This pathway primarily activates the IRF3/NF-κB signaling cascade upon recognition of cytosolic DNA, leading to the production of IFN-β, which subsequently promotes immune cell activation and tumor antigen presentation (Dvorkin et al., 2024). In this study, EPE significantly increased the phosphorylation levels of STING and IRF3 in NK-92 cells and promoted IFN-β release. Furthermore, the use of the STING inhibitor H151 notably inhibited the EPE-induced IFN-γ secretion, confirming that EPE primarily promoted NK cell activity by targeting the activation of the cGAS-STING pathway. This finding has dual significance: firstly, it demonstrates that traditional herbal compounds can directly affect innate immune signaling pathways, providing a model for the development of natural products as immune adjuvants; secondly, although the primary effector cells of the cGAS-STING pathway are dendritic cells or macrophages, recent studies have shown that activation of STING targets can also enhance the activity of NK cells and CD8^+^ T cells (Sen et al., 2019). Activation of the STING signaling pathway can significantly promote interferon release and enhance the infiltration of CD8^+^ T cells and NK cells in the tumor immune microenvironment. Interestingly, multiple studies have confirmed that Icaritin, the classic active compound of EPE, can promote CD8^+^ T cell infiltration and enhance the anti-tumor immune effects of PD-1 inhibitors (Li et al., 2022; Xu et al., 2024). However, as an emerging immune cell in anti-tumor immunity, the effect of EPE on NK cells has not yet been explored. This study expands the role of the cGAS-STING pathway in regulating NK cell function, suggesting its broad potential in immune modulation. After confirming that EPE promotes the release of type I interferons and activates NK cells through the cGAS-STING pathway, we combined High Performance Liquid Chromatograph (HPLC) screening to select the higher-content compounds of EPE. Molecular docking analysis was performed to examine the binding of these compounds with the STING protein, and it was found that the compounds of EPE exhibited strong binding capabilities with STING, with Epimedin C showing the strongest binding affinity.

Currently, multi-drug combination regimens for cancer therapy are progressively being widely implemented in clinical practice. Enhancing anti-tumor immune activity represents a critical research direction, particularly for addressing immune-suppressed “cold” tumors. For instance, recent studies have revealed that the tyrosine kinase inhibitor anlotinib can potentiate the efficacy of anti-PD-1 therapy by modulating the tumor microenvironment and enhancing CD8^+^T cell infiltration (Sha et al., 2024). However, this study demonstrates that EPE can activate NK cells through STING pathway activation. Furthermore, since STING activation is also known to promote T cell infiltration, it is plausible to hypothesize that EPE may synergize with PD-1/PD-L1 inhibitors to exert anti-tumor effects. Additionally, while EPE is a traditional Chinese medicine with documented idiosyncratic hepatotoxicity, previous studies have identified Icariside II, as its primary active compound (Wang et al., 2020). Consequently, the concentration and dosage in the development of this preparation can be managed and controlled, indicating promising translational potential.

## 5 Conclusion

In summary, this study reveals the potential of EPE in inhibiting HCC growth by activating NK cell through the cGAS-STING signaling pathway. This not only enriches the pharmacological profile of EPE but also provides new insights into the immune regulatory mechanisms of other tonic traditional Chinese medicines. As a natural STING agonist targeting NK cell activation, EPE may offer better safety and synergistic effects from its multi-compound composition. However, further in-depth exploration is needed to fully understand the specific mechanisms, active compounds, and clinical application prospects of *Epimedium*. This research may provide a new immunotherapeutic strategy for HCC patients and promote the use of traditional plants in modern cancer treatment.

## Data Availability

The datasets presented in this study can be found in online repositories. The names of the repository/repositories and accession number(s) can be found in the article/Supplementary Material.

## Supplementary material

SUPPLEMENTARY FIGURE S1 The compounds of EPE was identified using HPLC.

## Abbreviations:

CAR-NK, chimeric antigen receptor natural killer; CARs, chimeric antigen receptors; CD107a, Lysosomal-associated membrane protein 1; CETSA, cellular thermal shift assay; DARTS, drug affinity responsive target stability; EPE, *Epimedium* extract; FBS, fetal bovine serum; PBMCs, HCC,hepatocellular carcinoma; ICD, immunogenic cell death; IFN-β, interferon-β; IFN-γ, Interferon-γ; IRF3, Interferon Regulatory Factor 3; MDSCs, myeloid-derived suppressor cells; MS, mass spectrometry; NK cell, Natural Killer cell; Peripheral blood mononuclear cells; STING, Stimulator of Interferon Genes; TME, tumor microenvironment; TNF-α, tumor necrosis factor-α; Tregs, regulatory T cells.
