# Supplementary figures and images for: Epimedium brevicornu Maxim. extract activates natural killer cells against hepatocellular carcinoma via the cGAS-STING pathway
Source: Front Pharmacol. 2025 Nov 21;16:1681650. doi: 10.3389/fphar.2025.1681650 (PMC12678249; doi:10.3389/fphar.2025.1681650)

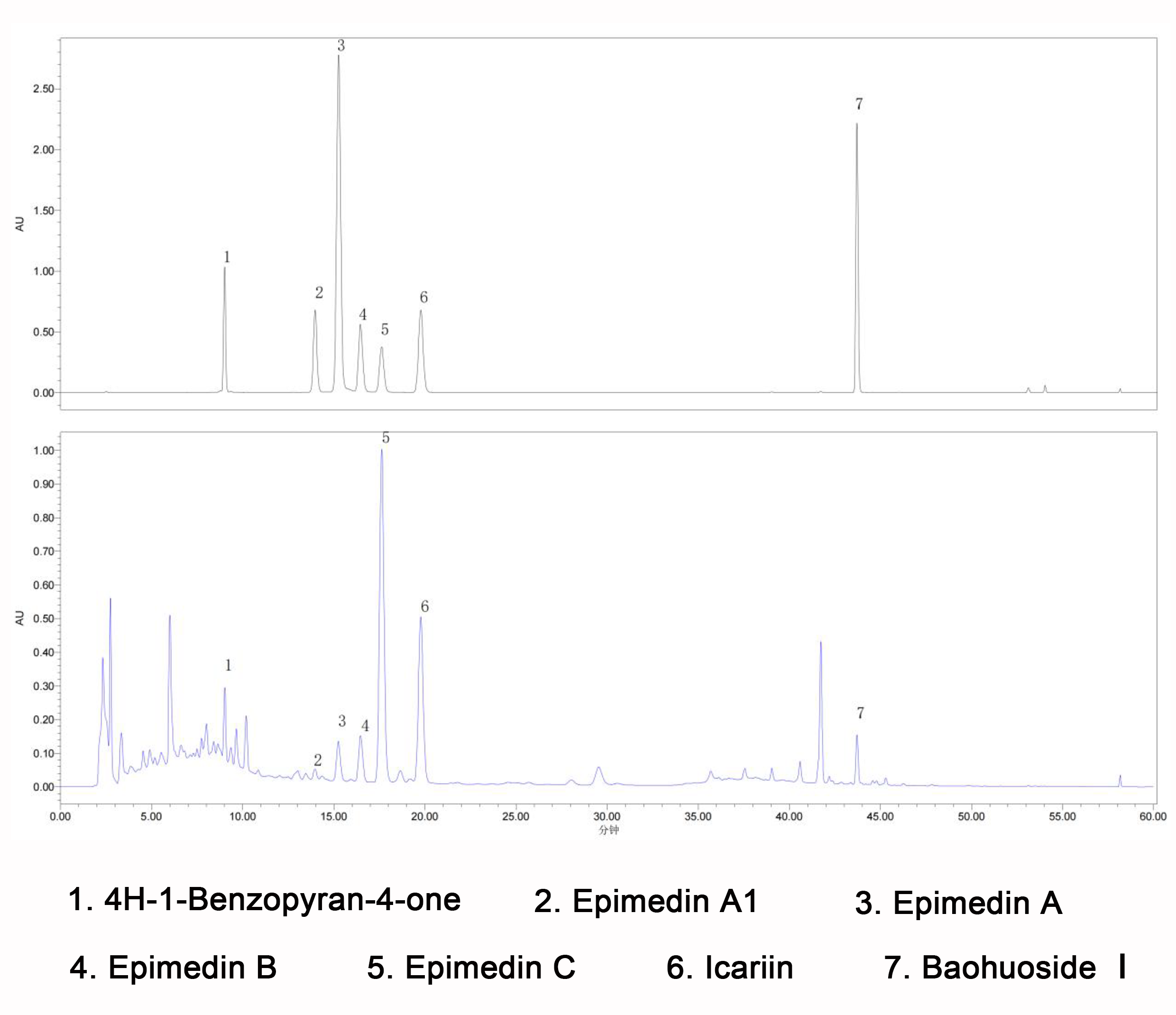

Supplement: Supplementary file 1 [file Image1.tif]
